# Accuracy of axillary ultrasound in preoperative nodal staging of breast cancer - size of metastases as limiting factor

**DOI:** 10.1186/2193-1801-2-350

**Published:** 2013-07-29

**Authors:** Angrit Stachs, Katja Göde, Steffi Hartmann, Bernd Stengel, Ulrike Nierling, Max Dieterich, Toralf Reimer, Bernd Gerber

**Affiliations:** Department of Gynecology and Obstetrics, University of Rostock, Südring 81, Rostock, 18059 Germany; Department of Neurology and Psychotherapy, University of Rostock, Rostock, 18146 Germany; Institute of Pathology, Südring 81, Rostock, 18059 Gemany

## Abstract

Since the performance of surgical procedures of the axilla in the treatment of early breast cancer is decreasing, the role of axillary ultrasound (AUS) as staging procedere has newly to be addressed. The aim of this study was to determine which patient or histopathological characteristics are related to false-negative AUS. In a retrospective study design data of 470 women with primary breast cancer were collected from patient charts and imaging and pathology records were reviewed. True positive and false negative axillary ultrasound groups were compared in terms of tumor size, histological subtype, grade, estrogen receptor (ER) and HER2 status, proliferation index, number and size of nodal metastases, extracapsular extension (ECE) and lymphovascular invasion (LVI). Of 470 patients, 166 (35%) were node positive, 79 of them with suspicious AUS. Factors associated with false negative AUS by univariate analysis were included in a multivariate model. By multivariate analysis, only size of nodal metastases was an independent factor for false negative AUS. In the sentinel lymph node biopsy (SLNB) subgroup, 45% of patients had nodal metastasis size less than or equal to 5 mm. In conclusion, AUS in preoperative staging of early stage breast cancer is limited by small size of metastases in a substantial number of patients. Prospective studies have to show whether small metastatic deposits leaving in patients in case of no axillary surgery have no negative effect on disease free and overall survival.

## Introduction

During the last decades, axillary lymph node metastases have been one of the most important prognostic parameters in patients with breast cancer. Today, in clinically negative axilla surgical (cN0) staging with sentinel lymph node biopsy (SLNB) represents the standard of care. However, more than 60% of all primary breast cancers do not have lymph node metastases. Due to the introduction of national screening programs a greater proportion of breast cancers are detected in an early stage with an increasing number of nodal negative disease. For these patients, even SLNB represents an overtreatment and may not be indicated. For many solid tumours the role of lymph node dissection is yet controversial, since it does not influence mortality. It is commonly acknowledged that the risk of developing metastases depends mainly on the biological behavior of the primary (seed and soil theory) (Engel et al. 2006). Moreover, a series of carefully performed prospective randomized trial focusing on axillary surgery in breast cancer exist showing a high rate of locoregional control achieved with multimodality therapy, even without axillary lymph node dissection (ALND) (Fisher et al. 2002; Group et al. 2006; Martelli et al. 2005; Giuliano et al. 2011). In fact, with the increasing influence of breast cancer biology on adjuvant treatment decisions, the relevance of nodal status is decreasing. It arises the question whether the information is necessary which we gain from identifying and examining the sentinel node (Gerber et al. 2011). Two planned prospective trials are focussing on this topic: SOUND (Sentinel Node vs. Observation after axillary Ultrasound) and German/Austrian INSEMA-Trial, an Intergroup study to compare axillary SLNB vs. no axillary surgery in patients with early primary breast cancer (Gentilini & Veronesi 2012). In this context, the role of preoperative axillary ultrasound (AUS) as a staging procedere has newly to be addressed. There is no doubt, that AUS, carried out by an experienced examiner, provides valuable information in the diagnosis of axillary metastatic involvement. But there are no standards defining sonographically suspicious lymph nodes. In a systematic review including 16 studies using morphologic criteria for positivity, sensitivity ranged from 26.4% (95% confidence interval [CI] 15.3-40.3%) to 75.9% (56.4-89.7%), and specifity varied between 88.4% (82.1-93.1%) and 98.1% (90.1-99.9%). Combining AUS with sonographically guided fine-needle aspiration (AUS-FNA), sensitivity varied between 30.6% (22.5-39.6%) and 62.9% (49.7-74.8%) and specificity reached nearly 100% (94.8-100%) (Alvarez et al. 2006). A recent meta-analysis from Houssami et al. including 31 studies focusing on ultrasound guided core needle biopsy (UNB) in preoperative breast cancer staging showed an estimated sensitivity of 79.6% (74.1-84.2%) and specificity of 98.3% (97.2-99.2%). Subgroup analysis revealed that UNB provided more utility in women with average or higher underlying higher risk for node metastases (Houssami et al. 2011).

However, the aim of most recent studies dealing with AUS in breast cancer was to identify women with lymph node metastases (imaging N1[iN1]) to spare SLNB and refer them directly to ALND. In view of the potentially avoidance of axillary surgery in future, the aim of our study (primary objective) was to identify factors influencing accuracy of AUS in preoperative breast cancer assessment. For that reason, we analyzed the sonographically missing metastatic lymph nodes (false negatives) at our institution. Secondary, we determined patients at risk for nodal involvement using tumour biological parameters as well as nomograms.

## Materials and methods

A total of 470 patients with primary breast cancer referred to our university hospital between February 2008 and January 2010 were enrolled in this retrospective study. In concordance to the institutional policy breast ultrasound including AUS was carried out by one of five experienced examiners before core needle biopsy. Lymph nodes were identified as abnormal according to sonographic criteria including absence of a fatty nodal hilum or a round hypoechoic node. Patients with sonographically negative nodes were subjected to SLNB. Patients with sonographically positive lymph nodes or contraindications for SLNB underwent ALND. Secondary, completion ALND was carried out in patients with positive sentinel lymph nodes. Patients with neoadjuvant chemotherapy were excluded from this analysis. The institutional review board approved the study and informed consent was obtained from all patients.

Patient charts were reviewed for patient demographics, primary tumour histology, tumour size, grade, hormone receptor status, HER2 status, results of AUS, number of sentinel lymph nodes (removed and involved), number of lymph nodes after ALND, number of positive nodes by histological examination, presence of lymphovascular invasion (LVI) or extracapsular extension (ECE). For determination of the size of the largest metastatic deposit of involved lymph nodes histological H&E slides were reviewed by our pathologist.

Statistical analysis was carried out using the SPSS 19.0 software package (IBM Ehningen, Germany). Sensitivity, specificity, positive predictive value, negative predictive value, and accuracy of AUS in the detection of lymph node metastases were calculated. To compare the proportion of missed axillary metastases between subgroups, Fisher’s exact (two variables), Pearson chi-square (three or more nominal variables or linear-by-linear association tests (three or more ordered variables) were used. The variables that were significant by univariate analysis were tested by multivariate logistic regression, to assess which of them had independent significance. A p-value <0.05 was considered statistically significant. All tests were two-sided. Clinical data were incorporated into the nomogram of the Memorial Sloan-Kettering Cancer center (MSKCC) to predict probability of SLN metastases (Bevilacqua et al. 2007). Discrimination of MSKCC nomogram was analyzed using receiver-operating characteristic (ROC) curve.

## Results

### Lymph node metastases and primary tumour pathology

**Figure 1:**
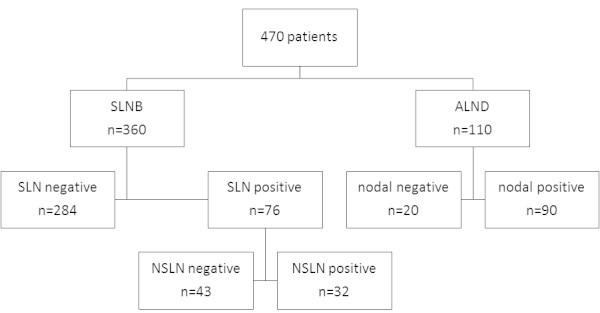
**Flow chart of involvement of axillary lymph nodes (n = 470).***ALND* axillary lymph node dissection, *SLNB* sentinel lymph node biopsy, *SLN* sentinel lymph node, *NSLN* nonsentinel lymph node.

From 470 patients with primary breast cancer, 166 patients (35.3%) had lymph node metastases. Baseline characteristics in relation to lymph node status are presented in Table 1. Concerning the surgical approach, 110 patients were primary treated with ALND. In 92 of them AUS was positive and 18 patients had contraindications for SLNB (large tumour size, previous extensive breast surgery). The remaining 360 patients underwent SLNB, 76 of them (21.1%) had metastatic involved lymph nodes. In 75 patients with pN + (sn) completion ALND was performed with the result of 32 patients having positive non-SLN (Figure 1).

**Table 1:** **Patient characteristics (n = 470)**

	Patients	pN+	%	p
**Age (years)**				n.s.
≤50	74	28	37.8	
>50	396	138	34.8	
**BMI**				n.s.
<25	180	60	33.3	
25-29.9	165	59	35.8	
≥30	124	46	37.1	
**Tumour stage**				<0.001
pT1	278	62	22.3	
pT2	164	81	49.4	
pT3 & pT4	28	23	82.1	
**Histological subtype**				0.045
Ductal	340	128	37.6	
Lobular	44	16	36.4	
Others	86	22	25.6	
**Grading**				<0.001
G1	67	6	9	
G2	261	88	33.7	
G3	142	72	50.7	
**Lymphangiosis**				<0.001
No	278	33	11.9	
Yes	192	133	69.3	
**Growth pattern**				<0.001
Unifocal	427	139	32.6	
Multicentric	40	25	62.5	
**ER status**				n.s.
Positive	383	129	33.7	
Negative	87	37	42.5	
**PR status**				0.024
Positive	339	109	32.2	
Negative	131	57	43.5	
**HER2 status**				n.s.
Negative	432	150	34.7	
Positive	38	16	42.1	
**Ki-67**				<0.001
≤14%	161	38	23.6	
>14%	282	122	43.3	
**Total**	**470**	**166**	**35.3**

**Figure 2:**
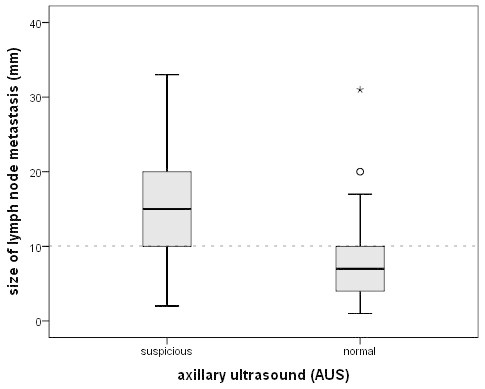
**Boxplot graph illustrating the difference in nodal metastasis size (mm) in all patients with suspicious (TP = true-positive) and normal axillary ultrasound (FN = false-negative).** In the TP group mean metastasis size is 15.5 mm (SD 6.80) in comparison to a mean size of 7.7 mm (SD 5.2) in the FN group mean (p < 0.001). At a cut-off of 10 mm metastasis size, approximately 75% of patients with lymph node metastasis ≥10 mm are detected with AUS, whereas 75% of patients with metastases <10 mm had normal AUS findings.

Axillary US was abnormal in 79 patients with metastatic lymph nodes and in 13 patients without nodal involvement. Sensitivity, specificity, positive predictive value, negative predictive value and accuracy of axillary US were 47.6%, 95.7%, 85.9%, 77% and 78.7%, respectively (Table 2). The proportion of sonographically missed axillary metastases was significant lower in large-sized tumours, grade 3 tumours, presence of lymphangiosis, ER/PR negative tumours, HER2-positive tumours and Ki-67 > 14% (Table 3) as well as nodal metastasis size >5 mm, N2- or N3-disease and extracapsular extension (Table 4). No differences in the false-negative AUS findings were seen according to age, BMI, histological subtype and multifocal/multicentric disease. To evaluate which of the parameters had independent prognostic value in the prediction of false-negative AUS, the factors that were significant by univariate analysis were tested in a multivariate model. By multivariate logistic regression, pathological size of nodal metastases was the only significant parameter associated with false negative ultrasound findings (Table 5)*.* According to the study group, from 166 patients with nodal involvement, lymph node metastasis size was available in 163 patients. As shown in Table 4, 41 patients (25.2%) had metastases ≤5 mm, which were detected with AUS in only in 4 cases (9.8%). In contrast, from 76 (45.8%) patients with lymph node metastases >10 mm, 55 (72.4%) were identified by ultrasound (Figure 2).

**Table 2:** **Comparison of axillary lymph node status as assessed with pathology and axillary ultrasound**

Axillary ultrasound	SLNB/ALND		Total
	Positive	Negative	
**Positive**	79	13	92
**Negative**	87	291	378
**Total**	166	304	470

Sensitivity, specificity, positive predictive value, negative predictive value and accuracy of axillary ultrasound in the detection of lymph node metastases: 47.6% (95%CI 40.1; 55.2), 95.7% (92.8; 97.5); 85.9% (77.3; 91.6); 77% (72.5; 80.9) and 78.7% (74.5; 82.9). *SLNB* Sentinel lymph node biopsy, *ALND* Axillary lymph node dissection, *CI* Confidence interval.

**Table 3:** **False-negative rate of axillary ultrasound (AUS) in different subgroups of 166 nodal-positive patients**

	pN+	AUS positive	AUS negative		p
		n	n	%	
**Age (years)**					n.s.
≤50	28	16	12	42.9	
>50	138	63	75	54.3	
**BMI**					n.s.
<25	60	28	32	53.3	
25-29.9	59	24	35	59.3	
≥30	46	26	20	43.5	
**Tumour stage**					0.001
pT1	62	20	42	67.7	
pT2	81	42	39	48.1	
pT3 & pT4	23	17	6	26.1	
**Histological subtype**					n.s.
Ductal	128	66	62	48.4	
Lobular	16	5	11	68.8	
Others	22	8	14	63.6	
**Grading**					0.005
G1	6	1	5	83.3	
G2	88	34	54	61.4	
G3	72	44	28	38.9	
**Lymphangiosis**					0.001
No	33	7	26	78.8	
Yes	133	72	61	45.9	
**Growth pattern**					n.s.
Unifocal	139	65	74	53.2	
Multicentric	25	14	11	44.0	
**ER status**					0.024
Positive	129	55	74	57.4	
Negative	37	24	13	35.1	
**PR status**					0.014
Positive	109	44	65	59.6	
Negative	57	35	22	38.6	
**HER2 status**					0.007
Negative	150	66	84	56	
Positive	16	13	3	18.8	
**Ki-67**					<0.001
≤14%	38	9	29	76.3	
>14%	122	69	53	43.4	
**Total**	**166**	**79**	**87**	**52.4**	

n.s. = not significant.

**Table 4:** **False-negative rate of axillary ultrasound (AUS) depending on extension of nodal involvement (n = 166)**

	nodal-positive	AUS positive	AUS negative		p
	n	n	n	%	
Nodal metastasis size *					<0.001
≤5 mm	41	4	37	90.2	
5.1-10 mm	46	19	27	58.7	
>10 mm	76	55	21	27.6	
**Number of metastatic involved lymph nodes**					<0.001
N1 (1–3)	86	27	59	68.6	
N2 (4–9)	48	28	20	41.7	
N3 (≥10)	32	24	8	25.0	
**Capsular infiltration**					<0.001
No	83	23	60	72.3	
Yes	83	56	27	32.5	

* 3 missing value.

**Table 5:** **Significant predictors of false-negative axillary ultrasound (false-negative ratio = OR) in 470 patients with breast cancer according to univariate and multivariate logistic regression**

	Univariate		Multivariate
	p-value	OR (95% CI)	p-value
**Tumour stage**			
T1	0,004	1.55 (1.17-2.04)	n.s.
T2-4		1	
**Grading**			
G1/2	0.003	1.61 (1.16-2.24)	n.s.
G3		1	
L**ymphangiosis**			
No	0.001	1.72 (1.33-2.22)	n.s.
Yes		1	
**ER status**			
Positive	0.024	1.63 (1.03-2.59)	n.s.
Negative		1	
**PR status**			
Positive	0.014	1.54 (1.08-2.22)	n.s.
Negative		1	
**HER2 status**			
Negative	0.007	2.99 (1.067-8.36)	n.s.
Positive		1	
**Ki-67**			
≤14%	<0.001	1.76 (1.34-2.30)	n.s.
>14%		1	
**Size of nodal metastasis**			
≤10 mm	<0.001	2.66 (1.81-3.91)	0,001
>10 mm		1	
**Nodal stage**			
N1	<0.001	1.96 (1.41-2.73)	n.s.
N2-3		1	
**Capsular infiltration**			
No	<0.001	2.22 (1.59-3.11)	n.s.
Yes		1	

OR = Odds ratio; CI = confidence interval; n.s. = not significant.

### Subgroup: patients with SLNB

**Figure 3:**
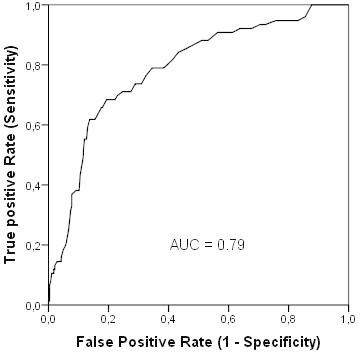
**Receiver-operating characteristic (ROC) curve calculation for the MSKCC nomogram applied to the sentinel cohort of our study population (n = 360).** The predictive accuracy of this model, as measured by the area under ROC curve (AUC) was 0.79 (95%CI 0.73; 0.84).

The mean age of the 360 patients operated with SLNB was 63 (range, 29–90) years, and the mean tumour size was 17.6 (range, 1–68) mm. Patients characteristics and tumour pathologic features are presented in Table 6. In total, 76 (21.1%) of 360 patients were identified with pN + (sn) status. Univariate analysis revealed that tumour size (>10 mm), a higher grading, presence of lymphangiosis and multicentric tumour growth were associated with positive nodal disease. In multivariate logistic regression analysis tumour size and multicentric growth were independent parameters related to a positive nodal status (Table 7). Application of the MSKCC nomogram to our sentinel cohort revealed a ROC value of 0.79 (Figure 3).

**Table 6:** **Patients with sentinel lymph node biopsy (n = 360)**

	Patients	pN + (sn)	%	p-value
**Age (years)**				
≤50	58	13	22.4	n.s.
>50	302	63	20.9	
**BMI**				
<25	146	31	21.2	n.s.
25-29.9	125	26	20.8	
≥30	89	19	21.3	
**Tumour stage**				
pT1a/b	64	7	10.9	0.009
pT1c	182	32	17.6	
pT2	111	37	33.3	
**Histological subtype**				
Ductal	259	57	22	n.s.
Lobular	31	8	25.8	
Others	70	11	15.7	
**Grading**				
G1	63	5	7.9	0.014
G2	211	48	22.7	
G3	86	23	26.7	
**Lymphangiosis**				
No	250	21	8.4	<0.001
Yes	110	55	50	
**Growth pattern**				
Unifocal	335	65	19.4	0.021
Multicentric	22	9	40.9	
**ER status**				
Positive	301	64	21.3	n.s.
Negative	59	12	20.3	
**PR status**				
Positive	270	57	21.1	n.s.
Negative	90	19	21.1	
**HER2 status**				
Negative	336	73	21.7	n.s
Positive	24	3	12.5	
**Ki-67**				
≤14%	142	24	16.9	0.067
>14%	194	47	24.2	
**Total**	**360**	**76**	**21.1**

**Table 7:** **Predictors of Sentinel Lymph Node metastases in 360 patients with breast cancer according to univariate and multivariate logistic regression**

	Univariate		Multivariate
Variable	p-value	Metastasis rate ratio (95%CI)	p-value
**Tumour stage**			
T1		1	
T2	0.002	2.05 (1.38-3.03)	0.019
**Grading**			
G1/2		1	
G3	0.014	1.38 (0.9-2.11)	n.s.
Lymphangiosis*			
No		1	
Yes	<0.001	5.95 (3.8-9.33)	
**Growth pattern**			
Unifocal		1	
Multicentric	0.021	2.1 (1.22-3.65)	0.051
**Ki-67**			
≤14%		1	
>14%	0,067	2.17 (1.05-4.5)	n.s

* Multivariate analysis included all preoperatively known parameters with significant results in univariate calculation (excluding lymphangiosis); n.s. = not significant.

**Figure 4:**
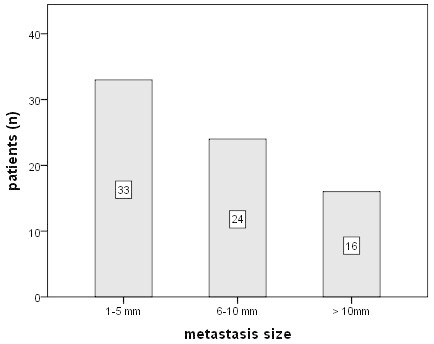
**Metastasis size in SLN-positive patients.** In 33/73 patients (45.2%) histological metastasis size was maximal 5 mm, 13 of them had micrometastases ≤ 2 mm.

To evaluate the tumour burden of patients with positive SLNB, we analyzed number of positive lymph nodes and size of largest metastastic deposit after completion ALND. Of 76 patients with positive lymph nodes after SLNB, one patient declined further axillary surgery (n = 75). Information about pathological size of lymph node metastases was available in 73 patients. Thirteen (17.8%) patients revealed only micrometastases (pN1mi, ≤ 2 mm), N1 disease (1–3 involved lymph nodes) was present in 55 (72.4%) patients, N2 disease (4–9 metastatic nodes) in 16 (21%) and N3 disease (≥ 10 metastatic nodes) in 5 (6.6%) patients. The mean size of largest metastatic deposit in patients with positive SLN was 7 (range, 1–31, median 6) mm. Metastatic deposits ≤ 5 mm were found in 33 of 73 patients (45%), whereas 16 patients (21.9%) had lymph node metastases > 10 mm (Figure 4).

**Figure 5:**
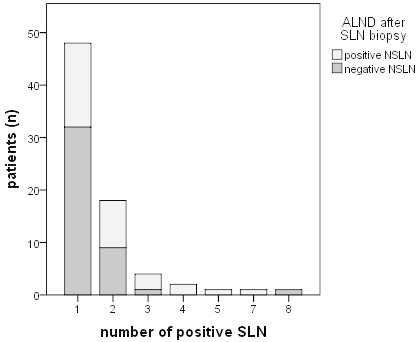
**SLN-positive patients after ALND (n = 75).** Involvement of non-SLN (NSLN). 43/75 patients (57.3%) with positive SLN had no further lymph node metastases. In patients with only one positive SLN the rate of positive NSLN is 33.3% (16/48).

A total of 43 (57.3%) from 75 patients with positive SLN had no further lymph node metastases (NSLN) at the time of completion ALND. In patients with only one positive SLN the rate of positive non-SLN was 33.3% (16/48) (Figure 5).

## Discussion

The role of preoperative AUS in early stage breast cancer is well-examined (Alvarez et al. 2006). However, AUS has a broad range of diagnostic perfomance and the experience of the examiner is crucial for diagnostic precision. The results of our study with sensitivity of 47.6% and specificity of 95.7% confirmed the unsatifactory sensitivity of AUS in axillary staging.

In an attempt to improve the results of AUS, numerous studies have been done dealing with fine needle aspiration (FNA) or core needle biopsy (CNB) of axillary lymph nodes in breast cancer patients. The meta-analysis of Houssami et al. 2397 including sonographically guides biopsies (FNA and CNB) of 4830 patients with a median prevalence of lymph node metastases of 47.2% showed a sensitvity of 75.0% and specificity of 98.5% (Houssami et al. 2011). However, as shown by a raw data analysis of the mentioned studies by Leenders sensitivities ranged from 6 to 63% if all patients were included and not only patients with suspicious AUS followed by FNA or CNB (Leenders et al. 2012). That means that addition of sonographically guided biopsy increases specificity and may help to identify patients with axillary lymph node metastases. But a negative FNA or CNB does not exclude lymph node metastases, since the proportion of false negatives reaches 37.1%.

In our study, the prevalence of lymph node metastases was 35.1% and nodal disease was associated with increasing tumour size, higher grading, presence of lymphangiosis, multicentric disease and high Ki-67 proliferation index. Accuracy of AUS reached 78.7%, but the rate of false negatives was considerable. There was no difference between several examiners (data not shown). Due to clinical experience it seems much more difficult to show lymph nodes sonographically in patients with markedly increased axillary fatty tissue. Unexpectedly, we did not found any difference in the false negatives depending on BMI.

To our knowledge, this is the first study showing the strong association between false negative AUS and size of lymph node metastases. Previous studies only differentiated between micro- and macrometastases and found a higher false negative rate in N1mi stage (Cools-Lartigue et al. 2013). Leenders et al. showed a sensitivity to detect micrometastases of 22.2% in comparison to a sensitivity to detect macrometastases of 51.9% (Leenders et al. 2012). But we must take into account the limits of ultrasound according to lesion size. In our study, 41/163 (25%) patients with N + disease had a maximum size of nodal metastases ≤5 mm. The false-negative rate in this subgroup reached 90%. This can partially be explained by the relatively poor ultrasound criteria defining suspicious lymph nodes used in this study. Other studies have shown a cortical thickness of ≥3 mm to be the most useful predictor of malignancy (Deurloo et al. 2003; Choi et al. 2009; Mainiero et al. 2010). However, the increase of sensitivity is connected with a decrease of specificity, which in clinical practise means that more patients are selected for ALND without having metastatic involved lymph nodes. On the other hands, there remains a considerable number of undetected metastatic involved lymph nodes also in these studies.

We have to ask the question whether other imaging techniques are able to detect small metastatic involved lymph nodes. A comparison between physical examination, mammography, ultrasound and magnetic resonance imaging (MRI) showed no advantage of MRI regarding the false negatives (Valente et al. 2012). Mortellaro et al. studied the specific parameters of MRI for axillary staging of breast cancer and found that only the presence of any axillary lymph node without a fatty hilum did correlate with axillary positivity (Mortellaro et al. 2009). With regard to the disadvantages including higher costs and patients physical restrictions there is no role for MRI in the routine use of preoperative axillary staging. The use of ^18^ F-fluorodeoxyglucose-positron emission tomography (^18^ F-FDG-PET) in combination with computed tomography (CT) to determine axillary nodal status is an active area of reserach (Peare et al. 2010). A recent study by Ueda et al. compared the ability of ^18^FDG-PET/CT with AUS and revealed a similar accuracy of both imaging techniques (Ueda et al. 2008). Actually, the performance of FDG-PET remains to low to replace assessment of axillary status by surgical biopsy and histological examination.

### Subgroup of patients with SLNB

Our study revealed metastatic involved SLN in 21.1% of patients, in subgroups of G1 tumours even 7.9% and tumour size ≤ 10 mm 10.8%. Multicentric disease and tumour size were independent risk factors for positive lymph nodes in multivariate analysis. Although SLNB is an extremely safe procedure with low morbidity, it has been suggested that patients with a low risk of axillary lymph node metastases should be spared SLNB (Viale et al. 2005). Concerning the multiparameter approach, Bevilacqua et al. from the Memorial Sloan-Kettering Cancer Center (MSKCC) developed a predictive model using nine preoperatively assessable variables associated with SLN metastasis, the so-called MSKCC nomogram. The diagnostic performance of this test was quite accurate with an area under the receiver operating characteristic (ROC) curve of 0.75 (Bevilacqua et al. 2007). The evaluation of this model in our study population as well as other cohorts confirmed the good results, but until now SLNB has proven as gold standard in axillary staging (Klar et al. 2009).

In this study cohort, the axillary tumour burden is low with 45.2% of pN + (sn) patients having a maximum size of lymph node metastases ≤ 5 mm and 43.3% having only one metastatic lymph node after completion ALND. Currently, there is an ongoing discussion about the need of completion ALND in pN + (sn) patients. According to the results of the ACOSOG Z0011 [Giuliano] trial, in patients with clinically negative axilla and one or two SLNs containing metastases treated with breast conserving therapy and tangential irradiation completion ALND can be omitted (Giuliano et al. 2011). Recent data of the IBCSG 23–01 trial showed no disadvantage in relapse-free and overall survival in patients with SLN micrometastases omitting completion ALND (Galimberti et al. 2013).

One step more would be to totally give up axillary surgery as staging procedure in clinically and sonographically negative axilla. From well-designed large studies dealing with safety of SLNB it is known that the rate of false negative SLNB is about 7 to 10% (Veronesi et al. 2003; Krag DN, Anderson SJ, Julian TB, Brown AM, Harlow SP, Ashikaga T, Weaver DL, Miller BJ, Jalovec LM, Frazier TG, Noyes RD, Robidoux A, Scarth HM, Mammolito DM, McCready DR, Mamounas EP, Costantino JP, Wolmark N & National Surgical Adjuvant Breast and Bowel Project Krag DN, Anderson SJ, Julian TB, Brown AM, Harlow SP, Ashikaga T, Weaver DL, Miller BJ, Jalovec LM, Frazier TG, Noyes RD, Robidoux A, Scarth HM, Mammolito DM, McCready DR, Mamounas EP, Costantino JP, Wolmark N & National Surgical Adjuvant Breast and Bowel Project ; Krag DN, Anderson SJ, Julian TB, Brown AM, Harlow SP, Ashikaga T, Weaver DL, Miller BJ, Jalovec LM, Frazier TG, Noyes RD, Robidoux A, Scarth HM, Mammolito DM, McCready DR, Mamounas EP, Costantino JP, Wolmark N & National Surgical Adjuvant Breast and Bowel Project 2007). But long time follow-up data of the NSAPB B-32 trial (SLNB + ALND vs. SLNB and ALND only in case of involved SLN) with 3986 patients and a mean follow-up time of 95.6 month have shown that there was no difference in overall survival, disease-free survival and number of recurrences in both study groups. Moreover, the rate of axillary node recurrence was markedly lower than expected. In detail, a total of 8 women with axillary node recurrence was seen in contrast to 57 expected cases of axillary recurrence in the group without ALND (n = 2011) with an underlying incidence of lymph node metastases of 29% and a false negative rate of 9.8% (Krag et al. 2010). Similar results were shown by Veronesi et al. with a cumulative incidence of axillary metastases of 1% at 5 years in 3548 patients with SLNB (Veronesi et al. 2009). However, the 5-year overall survival rate in this series was 98% with a high percentage of pT1 tumors and may not be representative for other studies. It remains the question: Can we accept false negative AUS for nodal metastases ≤10 mm in clinical practice? We know that pN + (sn) patients with low axillary tumour burden do not benefit from extensive axillary surgery in the era of sufficient local (tangential irradiation after BCS) and systemic adjuvant therapy. Moreover, the percentage of pN + patients is decreasing due to mammography screening programs.

## Conclusion

This study shows that accuracy of preoperative AUS in early stage breast cancer patients depends mainly on the size of axillary lymph node metastases. Metastatic deposits up to 10 mm represent a substantial number of false negative AUS and remain a diagnostic challenge. Otherwise, adjuvant therapy decisions become more and more independent of nodal involvement and recent studies showed no disadvantage in survival in case of potentially missing metastatic lymph nodes. Future prospective randomized studies including preoperative AUS (SOUND trial, INSEMA trial) will contribute to answer the question if surgical staging of the clinically and sonographically inconspicuous axilla is still necessary in early breast cancer treatment.
